# Special Issue: “Viruses Associated with Gastroenteritis”

**DOI:** 10.3390/v18010022

**Published:** 2025-12-23

**Authors:** Celso Cunha, Claudia Istrate

**Affiliations:** 1 Global Health and Tropical Medicine (GHTM), Institute of Hygiene and Tropical Medicine (IHMT-NOVA), NOVA University, Rua da Junqueira, 100, 1349-008 Lisbon, Portugal; 2 Interdisciplinary Center for Research in Animal Health (CIISA), Faculty of Veterinary Medicine, University of Lisbon, 1300-477 Lisbon, Portugal; claudiaistrate@fmv.ulisboa.pt

## 1. Introduction

Diarrheal diseases are the third leading cause of death in children under 5 years of age, with approximately 450.000 deaths per year, especially in low and middle income countries (LMICs). Yet, the burden of acute gastroenteritis (AGE) can be significantly reduced by vaccination campaigns and implementation of simple preventive sanitation measures. Despite the availability of four vaccines pre-qualified by WHO, of which two are recommended to be introduced in national immunization programs worldwide since 2009, rotaviruses remain the main cause of AGE with the highest burden in African sub-Saharan countries. Rotaviruses and noroviruses account for about two thirds of deaths caused by diarrhea in children under 5 years of age. However, other viruses, namely adenoviruses, sapoviruses, and astroviruses, may also have a non-negligible impact.

This Special Issue includes eight articles. The majority reports new data on the molecular epidemiology and genome evolution of rotavirus, norovirus, adenovirus, and sapovirus. Overall, the authors highlight the need for the implementation of full genome surveillance programs, continuous monitoring of emergence of new variants and strains, and their potential impact on the efficacy of currently available vaccines and influence on the design of future formulations.

## 2. Contributions

Vita et al. [1] present new data on the status, evolution and molecular epidemiology of rotaviruses in Angola (2021–2022), a country where epidemiological data is scarce, namely in the period after vaccine introduction, in 2014. In Brazil, Silva et al. [2] conducted a retrospective study (2012–2014) highlighting the emergence of new multiple reassortment events with rotaviruses commonly found in other continents, namely Africa and Asia. The detection of new variants and strains with potential to replace those currently circulating is stressed in both studies.

The importance of sustained genotyping and phylogenetic analysis of new isolates is also underlined for noroviruses. In Finland (2024/2025), Al-Hello et al. [3] report widespread high rates of detection of GII strains that cluster with other GII strains earlier detected in Europe and the United States. Moreover, Lee et al. [4] assessed norovirus detection rates in pre- and post-COVID-19 pandemic periods (2019–2024) in Korea. The most commonly found strains were also GII. However, a shifting genotype dynamic was detected in 2024. Both studies underscore the need for continuous surveillance with added importance in the context of norovirus vaccine development, and reinforcement of public health interventions.

The impact of high levels of expression of bacterial lipopolysaccharide (LPS) genes on norovirus infection was investigated in South Africa (2018–2020) by Kgosana et al. [5]. A positive correlation was found between high levels of expression of LPS and development of norovirus diarrhea in children aged between 3.5 and 9 months old. This observation may suggest an eventual role of bacterial LPSs and perhaps other components of the microbiome in the course and outcome of norovirus infections. This hypothesis deserves more comprehensive attention in a broader context of viral infections of the gastrointestinal tract.

The importance of monitoring circulating variants is further underlined for human enteric adenoviruses in a retrospective study conducted by Mello et al. [6] in Brazil (2021–2023). Sequencing of the hexon and/or fiber genes revealed that HAdV-F40/41 was the most frequently detected human enteric adenovirus genotype in stool samples from AGE cases. This genotype was also associated with higher viral loads when compared to non-enteric HAdVs.

Sapoviruses were investigated in a study by Guo et al. [7] using samples obtained from a viral diarrhea surveillance network in China (2022–2023). The overall detection rate was higher in children under 5 years of age (2.37%). Eight genotypes were identified by sequencing with the highest variation rates observed in the NS1 region of the genome.

At last, the work by Luchs et al. [8] spotlights an overlap of respiratory and enteric syndromes in children ≤ 3 years of age, in Brazil (2021). A search for different viruses was performed in 20 paired swab and stool samples from children with diarrheal and respiratory symptoms allowing detecting seven different virus families that may cause respiratory and enteric diseases: Human bocavirus, Norovirus, Human enteric adenovirus, Parechovirus, Enterovirus, Respiratory syncytial virus, and Influenza A virus. Human bocaviruses, which have been previously associated with both AGE and respiratory diseases, and noroviruses, were detected in 75% of cases, with co-infections observed in 65% of patients. Although limited by the small number of enrolled patients, the study points to the importance of implementation of accurate differential diagnosis procedures.

## 3. Outlook and Final Remarks

The burden of AGE remains unacceptably high, namely in children living in LMICs. Simple, and often low-cost sanitation measures, like provision of universal access to drinking water as well as the implementation of effective campaigns designed to improve hygiene and health literacy have a proven significant positive impact. Yet, these interventions should go hand in hand with effective vaccination campaigns. Apart from the aforementioned vaccines against rotaviruses, there are currently at least three norovirus vaccine candidates in different stages of development: a virus-like particle (VLP) formulation, a bivalent oral vaccine, and a trivalent mRNA vaccine. Even so, development of new and more effective formulations is only one of the many challenges that we face to reduce the burden of AGE. Raising awareness among policy decision makers for the urgent need to implement effective surveillance plans and preventive strategies is of utmost importance. The contributions contained in this Special Issue are certainly a drop in the ocean but the ocean continues to rise.

We thank all authors for sharing their high quality and impactful research as well as the reviewers whose selfless work were fundamental to maintaining scientific rigor and improved the quality of manuscripts. Finally, we express sincere gratitude to all members of the editorial staff for their commitment and professionalism since the beginning of the preparation of the Special Issue.
